# Homology-Independent Metrics for Comparative Genomics

**DOI:** 10.1016/j.csbj.2015.04.005

**Published:** 2015-05-04

**Authors:** Tarcisio José Domingos Coutinho, Glória Regina Franco, Francisco Pereira Lobo

**Affiliations:** a Departamento de Bioquímica e Imunologia, Programa de pós-graduação em Bioinformática, Instituto de Ciências Biológicas, Universidade Federal de Minas Gerais, Antonio Carlos Avenue, 6627, Belo Horizonte, Minas Gerais CEP 31270-901, Brazil; b Laboratório Multiusuário de Bioinformática, Embrapa Informática Agropecuária, André Tosello Avenue, 209, Barão Geraldo, Campinas, São Paulo CEP 13083-886, Brazil

**Keywords:** DOR, dinucleotide odds ratio, GC3S, frequency of G + C at the third position of synonymous codons, HI, homology-independent, HD, homology-dependent, ORF, open reading frame, RSCU, relative synonymous codon usage, NC, effective number of codons, NC′, effective number of codons considering GC3S, Comparative genomics, Homology-independent metrics, Genomic signatures

## Abstract

A mainstream procedure to analyze the wealth of genomic data available nowadays is the detection of homologous regions shared across genomes, followed by the extraction of biological information from the patterns of conservation and variation observed in such regions. Although of pivotal importance, comparative genomic procedures that rely on homology inference are obviously not applicable if no homologous regions are detectable. This fact excludes a considerable portion of “genomic dark matter” with no significant similarity — and, consequently, no inferred homology to any other known sequence — from several downstream comparative genomic methods. In this review we compile several sequence metrics that do not rely on homology inference and can be used to compare nucleotide sequences and extract biologically meaningful information from them. These metrics comprise several compositional parameters calculated from sequence data alone, such as GC content, dinucleotide odds ratio, and several codon bias metrics. They also share other interesting properties, such as pervasiveness (patterns persist on smaller scales) and phylogenetic signal. We also cite examples where these homology-independent metrics have been successfully applied to support several bioinformatics challenges, such as taxonomic classification of biological sequences without homology inference. They where also used to detect higher-order patterns of interactions in biological systems, ranging from detecting coevolutionary trends between the genomes of viruses and their hosts to characterization of gene pools of entire microbial communities. We argue that, if correctly understood and applied, homology-independent metrics can add important layers of biological information in comparative genomic studies without prior homology inference.

## 1 Introduction

Most computational methods available for comparative genomics rely on initial similarity searches to infer homology relationships and, consequently, analyze the wealth of genomic data currently available. Among others, current methods for comparative genomic analysis based on the detection of homologous sequences allow the 1) determination of the time of divergence between taxa through the theory of molecular clock [1]; 2) automatic annotation of new genomes based on orthology inference [2]; 3) estimation of the rates of evolution of protein families [3]; 4) analysis of the overall evolution of genomes through genome-scale analysis of patterns of gain/loss of genomic elements [4]; 5) searching for higher-order layers of positional genomic information (haplotypic blocks, synteny, etc.) [5]; and 6) genomic-scale search for patterns of positive Darwinian selection [6], among many others.

The computational methods for comparative genomic analysis based in the detection of homologous regions, from now on referred as homology-dependent (HD) methods, although crucial for several bioinformatic pipelines, are limited to genomic sequences with detectable homologous regions, usually identified through computationally intensive software that contains somewhat arbitrary cut-offs to define groups of homologous sequences [7,8]. The failure to detect such regions prevents the application of virtually any HD method and excludes several interesting classes of DNA sequences from further analysis. ORFans — orphans Open Reading Frames (ORFs) without any detectable similarity to other sequences — are commonly found in complete genomes and in environmental sequences and constitute a true “dark matter” of biological data that cannot be surveyed using traditional HD methods [9,10]. In some newly discovered taxa, such as the large DNA viruses from *Mimiviridae*, the vast majority of coding sequences do not share significant similarity with known proteins [11].

In this mini-review we compile a list of metrics used in comparative genomic studies that share an unusual property for this purpose: they do not rely on initial homology inference, and can be calculated from individual sequence data alone. Such metrics, from now on referred as homology-independent (HI) metrics, can be easily calculated for virtually any fragment of any genome that fulfills a few criteria, such as minimum length and complexity. These metrics usually detect biases by comparing the observed frequencies of nucleotide words, especially dinucleotide and codons, with expected frequencies for the same words. The dinucleotide usage patterns in a given genome are commonly referred in the scientific literature as genomic signatures, since they are also taxon-specific and highly conserved in a given genome [12–14]. Here we also highlight the relative strengths and weaknesses of such metrics and report comparative genomic studies that applied such metrics to extract biologically meaningful information that would be otherwise impossible to obtain using common HD comparative genomic methods.

## 2 Homology-Independent Metrics: Causes and Properties

### 2.1 Causes of Variation in Homology-Independent Metrics

Most explanations for the biased values observed in HI metrics are due to a complex interplay between three broad groups of phenomena that shape together the use of nucleotide words in genomes. One of these groups is composed of mutational pressures where a given nucleotide word is significantly more (or less) used than its expected frequency due to mutational events. Possible sources of mutational pressures are distinct transition/transversion ratios [15], CpG underrepresentation in vertebrate genomes due to methylation/deamination processes occurring in this dinucleotide [16] and distinct nucleotide incorporation efficiency by polymerases during genome replication [17], among many others.

A second group of phenomena responsible for HI biases in genomes is composed of selection pressure events, in which natural selection shapes the differential usage of nucleotide motifs. In fact, several nucleotide motifs (such as the “TATA box”) interact with the transcription/translation machinery and are classic examples of conservation of nucleotide words in genomic sequences due to selection pressure [18]. Another classic cause of variation in nucleotide words (codons) induced by selection pressure is the more-than-expected usage of synonymous codons that corresponds to the more abundant aminoacyl-tRNAs in cell cytoplasm in order to increase translation speed/efficiency, a selection pressure particularly strong in single-celled organisms [19] and in highly-expressed genes [20]. A final broad class of factors known to influence the use of nucleotide words in genomes is the occurrence of neutral processes such as genetic drift during the course of evolution [21]. Therefore, if properly modeled and interpreted, the results obtained through HI metrics in comparative genomic studies can highlight broad patterns of mutational and selective forces as well as random variations acting in the genomes under analysis.

### 2.2 General Properties of Homology-Independent Metrics

Besides not requiring previous homology relationships to analyze genomic data, HI metrics contain other general properties shared by most or all of them. An interesting property is that most HI metrics contain null models that take into account major factors already known to influence the frequencies of nucleotide words. Different HI metrics consider factors such as GC content, observed frequencies of smaller words that compose the word under analysis, degeneracy of the genetic code and amino acid usage, among others, when calculating null models. Therefore, any bias detected using HI metrics with proper null models is not explainable by factors already taken into account when computing null models, and represent biological phenomena that require further explanation/investigation. Also, several HI metrics are pervasive in the sense that values for whole genomes should persist at smaller scales. Some HI metrics remain reasonably constant for fragments with as few as 125 base pairs when compared with values calculated for entire genomes, or even when comparing coding and non-coding regions of genomes, making HI metrics a robust option to develop procedures to classify nucleotide sequences to taxonomic units, as in the case of genomic signatures [13].

Another useful aspect of HI metrics when applied to comparative genomics is the fact that some of them generate results that contain phylogenetic signal and are able to represent phylogenetic relationships, arguably with a more global view of the evolutionary process [22]. The patterns of DOR and codon usage bias in complete genomes of prokaryotes present a strong correlation with phylogenetic trees of 16S ribosomal RNA and housekeeping genes [14,23]. Comparative genomics using HI metrics may better reflect the global phylogenetic relationships between complete genomes by considering, for instance, events of horizontal transfer (HGT, one of the many factors known to change local frequencies of nucleotide words in genomic sequences) as part of the evolutionary signal in opposition to the reductionist analysis of single genes as proxies to faithfully represent the phylogenetic history of the entire genome.

## 3 Main Metrics for Homology-Independent Analyses

Several HI metrics have been used for comparative genomics. Supplementary Table 1 contains a non-exhaustive list of software to calculate HI metrics to allow users to analyze their own data. Below follows a formal description of the most common metrics:

### 3.1 Genomic Signatures

Genome signature is an umbrella term used to refer to similar concepts, but to different HI metrics. A genome signature refers to any HI metric that can be calculated from a DNA sequence with sufficient length and compositional complexity that enables the correct classification of the sequence to its source genome [12,24]. An ideal genomic signature should satisfy three major criteria: 1) it should be species-specific; 2) it should reflect phylogenetic history and 3) it should be pervasive [12].

#### 3.1.1 GC Content

The simplest known HI metric used as genomic signature is the GC content of genomic sequences (percentage of G + C in a sequence). Despite being a simple metric, GC presents a huge variation across genomes, ranging from approximately 20% in *Plasmodium falciparum* [25] to 70% in some actinobacteria [26]. GC content is reasonably constant within a given genome, and was already found to be correlated with several universal factors of microbial lifestyles such as temperature [27], niche complexity [28] and aerobiosis [29].

#### 3.1.2 Dinucleotide Odds Ratio

Dinucleotide Odds Ratio (DOR) is defined as the ratio between observed and expected frequencies of a dinucleotide in a sequence, and is perhaps the canonical example of both an HI metric and a genomic signature [24]. The expected frequency of a dinucleotide (null model) is defined as the product of the observed frequencies of its two nucleotides in the sequence under analysis, therefore removing background nucleotide frequency as a possible source of bias. DOR values close to one indicate observed frequencies close to expectation, and values significantly above or below one indicate the presence of mutational and/or selection bias actively shaping the frequency of dinucleotides. As arbitrary cutoff, DOR values observed outside the range of 0.78–1.25 are commonly considered to have low or high relative abundance, respectively [30]. Eq. (1) describes the DOR calculation for dinucleotide *xy* for a single-stranded sequence.

(1) $$P_{xy}=\frac{f_{xy}}{f_{x}f_{y}}\text{.}$$

Eq. (1) describes the calculation of dinucleotide odds ratio (*P_xy_*) for single-stranded genomes. *f_x_* and *f_y_* denote the frequency of mononucleotides *x* and *y* in a given sequence, and *f_xy_* denotes the observed frequency of dinucleotide *xy* in the same sequence.

The calculation shown in Eq. (1) is valid only for single-stranded sequences that do not obey the first Chargaff's parity rule. For double-stranded genomes the frequency of each nucleotide is calculated in a symmetrical way to take into account the complementary nucleotide located at the opposite strand. If we denote the nucleotide frequencies in double stranded genomes with “°”, f°(T) = f°(A) = (f(A) + f(T)) / 2 and f°(C) = f°(G) = (f(C) + f(G)) / 2. Based on the above equation and the first Chargaff's parity rule, the DOR for double-stranded sequences is calculated as follows:

(2) $$P_{xy}=\frac{2\left(f_{xy}f_{zw}\right)}{\left(f_{x}f_{y}\right)\left(f_{z}f_{w}\right)}\text{.}$$

Eq. (2) describes the calculation of dinucleotide odds ratio (*P_xy_*) for double-stranded genomes. *f_x_* and *f_y_* denote any two nucleotides, and *f_z_* and *f_w_* denote the frequencies of nucleotides *z* and *w*, complementary to *y* and *x*, respectively.

#### 3.1.3 Relative Synonymous Codon Usage (RSCU)

This metric is commonly used to estimate bias in the use of synonymous codons and removes the differences in the frequencies of amino acids as a possible bias factor (null model) [31]. Observed values are the counts of the *jth* codon for the *ith* amino acid, and expected values are calculated by counting the total of codons encoding the amino acid *i* in a given sequence divided by the degeneracy class of amino acid *i* (two, three, four or six). RSCU values for each codon are calculated according to Eq. (3):

(3) $$\mathrm{RSCUij}=\frac{X_{j}^{i}}{E_{j}^{i}}\text{.}$$

Eq. (3) describes the calculation of RSCU for codon *j*. *X*_*j*_^*x*^ — observed number of occurrences of the *jth* codon for the *ith* amino acid. *E*_*j*_^*i*^ — expected number of occurrences of *jth* codon for the *ith* amino acid. Expected values are calculated by counting all synonymous codons coding for amino acid *i* in a sequence divided by the number of synonymous codons that code for this amino acid (amino acid degeneracy class).

#### 3.1.4 Genomic Signatures Using Longer Words

Genomic signatures based on nucleotide words with length two and three are commonly used as HI metrics due to the immediate biological relevance of words of these lengths for pivotal biological processes involving nucleic acids, such as DNA modification mechanisms in vertebrates which recognize dinucleotides as modification sites [16] and translation, which is indissociable of the codon concept. However, using longer DNA words as genomic signatures adds more dimensions to compare and stratify sequence data (e.g. there are, respectively, 16, 64, 256 and 1024 different DNA words of lengths 2, 3, 4 and 5, respectively). Although not possessing a clear, intuitive biological relevance such as words of length two or three, the length increase arguably improves classification performance of genomic signatures metrics [32–35]. Null models for words of these lengths often involve more complex procedures, such as zero- or higher-order Markov models to account for the frequencies of smaller words that compose each DNA word [35].

### 3.2 Effective Number of Codons (NC) and Variations

#### 3.2.1 Effective Number of Codons (NC)

The effective number of codons (NC) is calculated for coding regions and represents the overall bias of preferential use of synonyms codons in a given gene/genome. The equation to calculate NC is conceptually similar to the calculation of the effective population size used in population genetics. It generates a single number that ranges from 20 (extreme codon usage bias where each amino acid is coded by only one codon) to 61 (absence of bias in the choice of synonyms codons, indicating equal usage for all codons) [36]. Extreme values of CG content in coding regions restrict the number of codons effectively available and, consequently, NC values are heavily influenced by CG content [37]. This metric is one of the most sensitive to detect biases in codon usage, and is calculated using the following equations (from [38]):

(4) $$\theta_{a}=\frac{n_{a}\sum_{i=1}^{k}p_{i}^{2}-1}{n_{a}-1}\text{.}$$

Eq. (4) describes the calculation of ***θ**_a_* (homozygosity of amino acid *a*). *p_i_* — frequency of codon *i*; *k* — number of synonymous codons for amino acid *a* (amino acid degeneracy class); *n_a_* — observed number of codons for amino acid *a* (only for amino acids with degeneracy class greater than one).

From the values of *θ_a_* for each amino acid one should compute the average values *θ_r_* for each amino acid degeneracy class (e.g. two, three, four or six-fold degeneracy) (Eq. (5)):

(5) $$\theta_{r}=\frac{1}{n_{RC}}\sum_{a\in RC}\theta_{a}\text{.}$$

Eq. (5) describes the calculation of average values ***θ**_r_* for each class of codons *r*. *n_RC_* — number of amino acids in a degeneracy class; *RC* — set of all amino acids belonging to a degeneracy class.

Finally, NC is computed as described in Eq. (6):

(6) $$NC=2+\left(\frac{9}{\theta_{2}}\right)+\left(\frac{1}{\theta_{3}}\right)+\left(\frac{5}{\theta_{4}}\right)+\left(\frac{3}{\theta_{6}}\right)\text{.}$$

Eq. (6) describes the calculation of NC. Each *θ* value in equation corresponds to an average value (*θ_r_*) calculated for each amino acid degeneracy class (two, three, four and six).

#### 3.2.2 NC-Plot

Since NC values are strongly influenced by GC content [36] it is useful to compare observed NC values against theoretical values calculated in function of GC content (null model) in order to exclude GC content as a possible source of variation. An approach in this direction is the calculation of NC plots, which consists of a theoretical curve correlating expected values of NC as a function of GC content at third bases of synonymous codons (GC3S). GC3S are commonly assumed to be a good proxy of “true” background genome composition regarding nucleotide frequencies since these positions are supposed to be under a more relaxed selection pressure (although there are controversies, see e.g. [39]). By including in the chart the observed values of GC3S and NC for a given coding sequence and finding points located above or below the theoretical curve it is possible to detect coding sequences with biased observed NC values after considering GC3S values. Eq. (7) generates the theoretical curve of NC plots:

(7) $$NC_{\mathrm{theoretical}}=2+f_{GC3S}+\left(\frac{29}{f_{GC3S}^{2}+{\left(1-f_{GC3S}\right)}^{2}}\right)\text{.}$$

Eq. (7) describes the theoretical values of NC as a function of GC3S. *f*_*GC*3*S*_ — frequency of GC3S. The resolution of this equation with *f*_*GC*3*S*_ parameter ranging from zero to one generates the theoretical curve of NC plot.

#### 3.2.3 Effective Number of Codons Considering the GC Content (NC′)

Another approach to deal with the strong influence of GC content over NC values was the development of a new metric, conceptually similar to NC, but that takes into account GC content, called NC′ [38]. Therefore, any bias evidenced by NC′ must be interpreted excluding GC content as a possible source of variation. However, other biases were introduced in this new metric. For instance, while NC values range from 20 to 61 and have clear and intuitive biological meaning, the NC′ values range from 0 to 61, which are not readily interpretable [40].

NC′ metric uses the chi-square test (*X*^2^) to calculate the deviation of observed frequencies of use of each codon *i* (*p_i_*, Eq. (8)) when compared with expected values (*e_i_*). Expected values may be calculated in various ways, such as from the frequencies of mono, di or trinucleotides comprising the codon, therefore accounting for distinct null model scenarios. The expected deviation value for each amino acid (*X*_*a*_^2^) is calculated as follows:

(8) $$X_{a}^{2}=\sum_{i=1}^{k}\frac{n_{a}{\left(p_{i}-e_{i}\right)}^{2}}{e_{i}}\text{.}$$

Eq. (8) describes the calculation of expected deviation values for each amino acid *a*. *i* — codon under analysis; *k* — amino acid degeneracy class; *n_a_* — number of observed codons for amino acid *a*; *p_i_* — observed frequency of codon *i*; *e_i_* — expected frequency of codon *i*.

Having the *X*_*a*_^2^ values one can compute *θ*_*a*_^'^ (conceptually similar to *θ_a_* used in NC calculation) as shown in Eq. (9):

(9) $$\theta_{a}^{'}=\frac{X_{a}^{2}+n_{a}-k}{k\left(n_{a}-1\right)}\text{.}$$

Eq. (9) describes the calculation of modified homozygosity values (***θ***_***a***_^'^) for amino acid *a*. *n_a_* — number of observed codons for amino acid *a*; *k* — amino acid degeneracy class.

From the values of *θ*_*a*_^'^ one can calculate NC′ in a manner similar to NC computation as described in Eqs. (5) and (6).

## 4 Current Applications of Homology-Independent Metrics in Comparative Genomics

The first and still the most popular field in comparative genomics with extensive application of HI metrics is the taxonomic classification of biological sequences or subsequences without prior phylogenetic tree reconstruction, such as in the case of genomic signatures. For instance, several pipelines for classification of sequences from environmental genomics to taxonomic space (binning procedures in metagenomics studies) rely on HI metrics [41–43]. Additionally, other classification methods to detect subsequences with biased distribution of HI metrics within longer genomic sequences could be used to detect several cases of exogenous DNA in a given genome. Several tools already make use of HI metrics for this purpose aiming at detecting important classes of evolutionary events, such as general cases of horizontal gene transfer (HGT) [44–46] and the detection of particular cases of HGT, such as genomic/pathogenicity islands [47] and phage integration sites [48]. For some groups of organisms such as large DNA viruses, HI metrics are sometimes the only class of tools available to study how these genomes evolved such large repertories of ORFans and to demonstrate that they arrived through multiple HGT events [11].

Nowadays, HI metrics have been used to answer questions in comparative genomics far beyond their initial use as genomic signatures for taxonomical classification of sequences. Such metrics were recently used to detect community-specific signatures of synonymous codon usage biases in metagenomic samples from different ecological niches. Such biased codons correlate with expression levels and occur regardless of individual phylogeny of organisms [49]. Additionally, such community-specific codon usage biases also predict lifestyle-specific genes, detecting coding sequences relevant for adaptation of organisms to specific ecological niches. These studies revealed a higher level of organization of metagenomic samples, where entire microbial communities share gene pools optimized for cross-genome translation and behave as a single meta-genome. Codon usage biases alone are also capable to predict other features for single microorganisms and/or microbial communities such as growth speed [50] and can be used to infer gene function based solely on the evolutionary changes for translation efficiency [51].

HI metrics have also been used to detect coevolutionary trends in biological systems composed of viruses and their hosts. The genomes of such disparate organisms coexist in the same cellular space and compete for the same resources. Therefore, it is reasonable to assume viral and host genomes to share some common compositional features due to constraints induced by host factors, such as the molecular mechanisms for the detection of foreign nucleic acids and for the translation of coding sequences.

The work of Lobo et al. 2009 used HI metrics (DOR and RSCU) to detect coevolutionary trends in a virus-host biological system [52]. The *Flaviviridae* family is composed of monophyletic viruses that infect vertebrate (mammals and birds) and/or invertebrate (ticks and mosquitoes) organisms. This work assumes that *Flaviviridae* that infect a single host lineage would be subjected to specific host-induced pressures and, therefore, selected by them. The authors observed that the two host groups possess very distinctive dinucleotide and codon usage patterns. A pronounced CpG under-representation was found in the vertebrate group, possibly induced by the methylation-deamination process, exclusive of vertebrate genomes, as well as a prominent TpA decrease. The invertebrate group displayed only a TpA frequency reduction bias, with no CpG bias. *Flaviviridae* viruses mimicked host nucleotide motif usage in a host-specific manner. Vertebrate-infecting viruses possessed under-representation of CpG and TpA, and insect-only viruses displayed only a TpA under-representation bias. Especially, single-host *Flaviviridae* members which persistently infect mammals or insect hosts (*Hepacivirus* and insect-only *Flavivirus*, respectively) were found to possess a codon usage profile more similar to that of their hosts than to phylogenetically related *Flaviviridae*. Vertebrates and mosquito genomes are under very distinct lineage-specific constraints, and *Flaviviridae* viruses which specifically infect these lineages appear to be subject to the same evolutionary pressures that shaped their host coding regions, evidencing lineage-specific coevolutionary processes between viral and host genomes that could not be surveyed using HD metrics.

## 5 Summary and Outlook

Since Karlin et al. 1994 described DOR and demonstrated its utility as a genomic signature [24,30], several compositional parameters calculated from nucleotide sequence data alone have been developed that fulfill and even surpass the original requisites to be classified as genomic signatures. Besides being used for their original purpose (classification of biological sequences to a taxonomic space), several other layers of biologically meaningful information are available through these metrics. In common, all these metrics are: 1) calculated from genomic sequence data alone; 2) do not rely on homology inference and 3) add a new layer of biologically meaningful data to the genomes under analysis, such as taxonomic information in the case of genomic signatures, but also other information such as putative gene expression profiles and convergent coevolutionary patterns.

The pervasiveness observed in HI metrics that made them useful for sequence classification into taxonomic space also permeates through other biological dimensions and could be used to describe and model biological entities in other classification systems besides biological taxonomy, with a utility far broader than the original scope of genomic signatures. For this reason, we argue that these metrics are better described as homology-independent (HI) metrics for comparative genomics. Genomic sequences from disparate groups that contain some niche superposition, such distinct species of microorganisms belonging to the same ecological community or viruses and their respective hosts coexisting in the same environment, were found to possess surprising patterns of coevolutionary events not known before HI metrics were applied to investigate them. The use of HI metrics as tools for comparative genomic analysis, if correctly understood and applied, can reveal important pieces of biological information in genomic sequences without priorhomology inference.

## Appendix A Supplementary Data

Supplementary Table 1 Examples of software commonly used to calculate HI metrics.
