# Supplementary material for: Homology-Independent Metrics for Comparative Genomics
Source: Comput Struct Biotechnol J. 2015 May 4;13:352–7. doi: 10.1016/j.csbj.2015.04.005 (PMC4446528; doi:10.1016/j.csbj.2015.04.005)
Supplement: Supplementary Table 1 — Examples of software commonly used to calculate HI metrics. [file mmc1.docx]

| Program (availability) | Metrics available | URL for use/download | Input/output | Reference |
| --- | --- | --- | --- | --- |
| CAIcal (web tool) | - G+C content  - RSCU | <http://genomes.urv.cat/CAIcal/> | fasta/table | [1] |
| GCUA (standalone software) | - RSCU  - NC | <http://bioinf.nuim.ie/gcua/> | fasta/table | [2] |
| ACUA (standalone software) | - G+C content  - RSCU | <http://www.bioinsilico.com/acua> | fasta/table | [3] |
| CodonW (standalone software) | - RSCU  - NC | <http://codonw.sourceforge.net/index.html> | fasta/table | [4] |
| EMBOSS geecee/compseq (standalone software, web tool) | - G+C content  - DOR  - odds ratio of DNA words up to length 6 | <http://emboss.sourceforge.net/> | many*/many* | [5] |

*gcg, embl, swissprot, fasta, ncbi, genbank, nbrf, codata, strider, clustal, phylip, acedb, msf, ig, staden, text, raw, asis (from <http://emboss.sourceforge.net/docs/faq.html#E>)

**Supplementary References**

1. Puigbo P, Bravo IG, Garcia-Vallve S (2008) CAIcal: a combined set of tools to assess codon usage adaptation. Biol Direct 3: 38.

2. McInerney JO (1998) GCUA: general codon usage analysis. Bioinformatics 14: 372-373.

3. Vetrivel U, Arunkumar V, Dorairaj S (2007) ACUA: a software tool for automated codon usage analysis. Bioinformation 2: 62-63.

4. Peden J (1997) CodonW. Trinity College.

5. Rice P, Longden I, Bleasby A (2000) EMBOSS: the European Molecular Biology Open Software Suite. Trends Genet 16: 276-277.
